# ASSOCIATION OF PLASMA LIPID METABOLITES WITH ANKLE-BRACHIAL INDEX IN THE LONG LIFE FAMILY STUDY

**DOI:** 10.1093/geroni/igac059.1557

**Published:** 2022-12-20

**Authors:** Allison Kuipers, Ryan Cvejkus, Bharat Thyagarajan, Gary Patti, Mary Feitosa, Jonas Mengel-From, Emma Barinas-Mitchell, Joseph Zmuda

**Affiliations:** University of Pittsburgh, Pittsburgh, Pennsylvania, United States; University of Pittsburgh, Pittsburgh, Pennsylvania, United States; University of Minnesota Twin Cities, MINNEAPOLIS, Minnesota, United States; Washington University, Department of Chemistry, Genetics, and Medicine, St. Louis, Missouri, United States; Washington University, St Louis, Missouri, United States; University of Southern Denmark, Odense, Syddanmark, Denmark; University of Pittsburgh, Pittsburgh, Pennsylvania, United States; University of Pittsburgh, Pittsburgh, Pennsylvania, United States

## Abstract

We tested the association of lipid metabolites with ankle-brachial index (ABI) in the LLFS. Minimum ABI from doppler was used. Participants underwent phlebotomy after >8 hours fasting. Plasma metabolites were isolated via solid phase extraction. Lipid metabolites (N=193) were measured using RPLC-MS and corrected for batch effects in 948 participants. We used linear mixed models adjusted for age, sex, site, fasting, and relatedness. Twenty metabolites were associated with ABI (FDR P<0.05), of which 17/20 were triacyclglycerol (TAG) species, plus cholesterol ester (22:6), phosphatidylcholine (35:7), and phosphatidylethanolamine (38:7). After additional adjustment for traditional ABI risk factors, only two TAGs (50:1, 42:0) were significantly associated after multiple testing correction. While not significant in the base model, the metabolite lysophosphatidylcholine (18:3) demonstrated a marginally significant protective association after full adjustment (P=0.014). While triglycerides are known correlates of atherosclerotic deposition, these findings may identify novel metabolic correlates of peripheral vascular health in long-lived individuals.

